# Modification of Microelectrode Arrays with High Surface Area Dendritic Platinum 3D Structures: Enhanced Sensitivity for Oxygen Detection in Ionic Liquids

**DOI:** 10.3390/nano8090735

**Published:** 2018-09-17

**Authors:** Ghulam Hussain, Anthony P. O’Mullane, Debbie S. Silvester

**Affiliations:** 1 Curtin Institute for Functional Molecules and Interfaces, School of Molecular and Life Sciences, Curtin University, GPO Box U1987, Perth 6845, Australia; ghulam.hussain1985@gmail.com; 2 School of Chemistry, Physics and Mechanical Engineering, Queensland University of Technology (QUT), Brisbane, Queensland 4001, Australia; anthony.omullane@qut.edu.au

**Keywords:** 3D nanostructures, electrodeposition, platinum, oxygen sensing, gas detection, microarrays, room temperature ionic liquids

## Abstract

Electrochemical gas sensors are often used for identifying and quantifying redox-active analyte gases in the atmosphere. However, for amperometric sensors, the current signal is usually dependent on the electroactive surface area, which can become small when using microelectrodes and miniaturized devices. Microarray thin-film electrodes (MATFEs) are commercially available, low-cost devices that give enhanced current densities compared to mm-sized electrodes, but still give low current responses (e.g., less than one nanoamp), when detecting low concentrations of gases. To overcome this, we have modified the surface of the MATFEs by depositing platinum into the recessed holes to create arrays of 3D structures with high surface areas. Dendritic structures have been formed using an additive, lead acetate (Pb(OAc)_2_) into the plating solution. One-step and two-step depositions were explored, with a total deposition time of 300 s or 420 s. The modified MATFEs were then studied for their behavior towards oxygen reduction in the room temperature ionic liquid (RTIL) [N_8,2,2,2_][NTf_2_]. Significantly enhanced currents for oxygen were observed, ranging from 9 to 16 times the current of the unmodified MATFE. The highest sensitivity was obtained using a two-step deposition with a total time of 420 s, and both steps containing Pb(OAc)_2_. This work shows that commercially-available microelectrodes can be favorably modified to give significantly enhanced analytical performances.

## 1. Introduction

Electrochemical methods have been widely investigated over several decades for the detection of chemical species dissolved in solution [1,2,3,4]. Typically, three (or sometimes two) electrodes are placed in ionic contact using a solvent containing a supporting electrolyte. A potential is applied between the working and counter electrodes, which induces a current proportional to the concentration of analyte. In recent years, there has been a drive towards the miniaturization of electrochemical cells and electrode designs, and various planar electrode devices have been commercially developed to address this need, with the working, counter, and reference electrodes printed in a small area (e.g., screen-printed electrodes, thin-film electrodes, and interdigitated electrodes) [5,6,7,8]. Several companies presently manufacture these commercially available devices (e.g., DropSens, MicruX, Zensor, Kanichi, Zimmer & Peacock), with various designs incorporating both mm- and micron-sized working electrodes. The use of microdisk electrodes for electroanalytical chemistry is particularly beneficial due to the higher current density, lower Ohmic drop contributions, and a smaller amount of electrode material required (especially important where expensive nobel metals are used), compared to conventional mm-sized surfaces. The overall current can be enhanced by employing arrays of microdisk electrodes [5,6,7,8,9,10,11,12], ideally sufficiently separated so that their diffusion fields do not overlap and each individual electrode can be addressed independently [11,12]. Room temperature ionic liquids (RTILs) are seen as favorable solvents for use with such miniaturized devices [13,14].

RTILs are a class of solvent that possess several archetypal properties, such as negligible vapor pressures, high thermal and chemical stability, wide electrochemical windows, high polarity, high viscosity, intrinsic conductivity, and good solvation abilities [15,16]. Their near-zero volatility requires only very small quantities of solvent (down to as low as micro or nano-litre quantities in some cases) [17,18,19,20], and they can be dropcast onto the planar electrodes of miniaturized devices. This is of huge benefit compared to traditional solvents (e.g., water or organic solvents), which can evaporate in a relatively short amount of time, particularly in hot and dry conditions. However, a drawback of RTILs is their high viscosity, which can lead to slower diffusion coefficients and lower currents. Arrays of microelectrodes can thus be employed with RTILs to improve current signals [13,14], but it is possible to further enhance their performance through improved electrode designs. We recently demonstrated this enhancement possibility by forming smooth, cauliflower shaped structures through electrodepositing platinum into the recessed holes of MATFEs, with overlapping diffusion zones roughly facing the adjacent electrodes [18]. These modified electrodes showed enhanced sensitivities for ammonia detection in ionic liquids [18], and these electrodes could be particularly beneficial for detecting highly toxic gases at low concentrations.

In the present work we aim to further improve the analytical performances of MATFEs, by varying the deposition parameters and composition of the plating bath to create dendritic structures resulting in even higher surface areas. The electrodeposition of Pt nanostructures with high surface area can be readily achieved by using approaches, such as hydrogen bubble dynamic plating [21] or introducing species into the electrolyte, which directs the growth of the deposit. For the latter, it has been shown that the inclusion of surfactant liquid crystals results in the fabrication of highly porous platinum [22]. The disadvantage of these approaches in the context of electrodeposition into recessed arrays of electrodes is that the hydrogen bubble dynamic plating method is more suitable to larger substrates, whilst the use of organic surfactants may result in surface contamination with species that could potentially interfere with the sensing process. Previous work has demonstrated that the inclusion of inorganic species in the electrolyte can also direct the growth of metal deposits, which alleviates the problem of surface contamination by organic moieties [23]. An example of this is lead acetate, which has been used for the formation of both gold and platinum nanostructured surfaces, which have been used for a variety of applications, including dopamine detection [24], gas phase mercury sensing [25], nitrite ion detection [23], methanol oxidation [26,27], and oxygen reduction [27]. These examples indicate that the inclusion of lead acetate does not affect the sensing properties of the electrodeposit, but rather enhances its performance due to the increased surface area from dendritic type structures, as well as the active sites generated when Pt is electrodeposited in this fashion. In this work, dendritic structures are produced and oxygen gas was chosen as a suitable analyte, to demonstrate the sensitivity enhancement offered by these modified microelectrode array surfaces.

## 2. Materials and Methods

### 2.1. Chemical Reagents

All chemicals were commercially available and used as received without further purification. Ethanol (EtOH, 99%), acetone (99%), sulfuric acid (98% *w*/*w* [18.4 M]) and zinc chloride (ZnCl_2_, 40% *w*/*v*, used as a soldering flux for connecting wires with electrodes), chloroplatinic acid hydrate (H_2_PtCl_6_ xH_2_O, trace metal basis, ≥99.9%), and lead acetate trihydrate (Pb(OAc)_2_ 3H_2_O, ≥99.9%), were purchased from Sigma-Aldrich (Castle Hill, NSW, Australia). Ferrocene (Fe(C_5_H_5_)_2_, 98% purity, Fluka) was used as received to prepare ferocene/ionic liquid mixtures. The room temperature ionic liquid (RTIL) octyltriethylammonium bis(trifluoromethylsulfonyl)imide ([N_8,2,2,2_][NTf_2_], >98%), was purchased from Merck, Kilsyth, Victoria, Australia. Ultrapure water with a resistance of 18.2 MΩ.cm was prepared using an ultrapure water purification system (Millipore Pty Ltd., North Ryde, NSW, Australia). Acetonitrile (MeCN, >99.8%, Fischer Scientific) was used for washing electrodes after use. High purity oxygen gas (>99.5%) was purchased from CAC gases (Auburn, NSW, Australia). Nitrogen gas (for dilution of O_2_) was obtained from a ≥99.99% high purity, compressed nitrogen cylinder (BOC gases, Welshpool, WA, Australia).

### 2.2. Electrochemical Experiments and Electrodeposition Parameters

All experiments were performed using a PGSTAT101 Autolab potentiostat (Metrohm Autolab, Gladesville, NSW, Australia) interfaced to a PC with Nova 1.11 software, at laboratory room temperature (294 ± 1 K), inside an aluminum Faraday cage present in the fume cupboard to reduce electrical interference. Platinum (Pt) microarray thin-film electrodes (MATFEs, MicruX Technologies, Oviedo, Spain, ED-mSE1-Pt) were used as the sensing device and used for further modification. These consisted of a 1 mm diameter Pt working electrode (thin-film of 150 nm thickness) on a Pyrex glass substrate covered with a layer of SU-8 resin, into which 90 micro-holes (µ-holes) of 10 µm diameter were made to create 90 recessed microdisk electrodes. The center-to-center distance of each μ-hole was 100 ± 1 μm (10× diameter), and the depth was 3 ± 0.5 μm. The planar electrode device had inbuilt Pt thin film counter and reference electrodes, deposited close to the working electrode, to delimit the electrochemical cell, and thus enable the use of very small sample volumes (e.g., 3 µL in this study).

Prior to electrodeposition experiments, the MATFEs were electrochemically activated in 0.5 M H_2_SO_4(aq)_, by scanning between + 1.40 and −0.24 V vs. an external Ag/AgCl (0.1 M KCl) reference electrode (BASi, West Lafayette, IN, USA) and Pt coil counter electrode (Goodfellow Ltd., Cambridge, UK), at a sweep rate of 500 mVs^−1^ for ca. 300 cycles. The electrodes were then washed with ultrapure water and dried under a nitrogen stream. The recessed μ-holes were decorated electrochemically with Pt-deposits of various shapes and geometries. The platinum plating solution consisted of 20 mM H_2_PtCl_6_ in N_2_-saturated 0.5 M H_2_SO_4_. The plating solution was also spiked with 2 mM Pb(OAc)_2_ to create 3D dendritic-shaped nanostructures. Dendritic structures find use in a number of research fields, including hybrid nanomaterials, flexible ceramics, biomaterials, and polymer composites [28,29]. During the deposition process, the following parameters varied: Total deposition time (300 s or 420 s), number of deposition steps (1 or 2), and composition of the platinum plating solution (with and without lead acetate). Electrodeposition involved holding at the open circuit potential (OCP, ~0.75 V), then applying an overpotential (−0.2 V) vs. a stable Ag/AgCl reference electrode, to produce instant nucleation and growth of Pt-deposits, as described in References [30,31,32,33,34]. During deposition, the plating solutions were stirred under strong magnetic stirring to ensure a fast rate of mass transfer, ensuring a constant rate of flux. The eight different electrochemical procedures that were employed, and their abbreviations used in this work, are given in Table 1.

The Pt-deposited MATFEs were electrochemically activated in N_2_-saturated 0.5 M H_2_SO_4(aq)_ by scanning the potential between +1.40 V and −0.24 V vs. Ag/AgCl (0.1 M KCl) and a Pt coil counter electrode, at a sweep rate of 500 mVs^−1^ for ca. 10 cycles to ensure there was no oxide layer, chloride ions (Cl^−^), or Pb contents adsorbed on the active sites, as described in Reference [35]. The electrochemical surface area (ESA) of the Pt-deposited MATFEs was calculated from the area under the hydrogen adsorption/desorption peaks using standard methods, explained in References [35,36].

### 2.3. Oxygen Gas Experiments

For gas sensing experiments, modified and unmodified MATFEs were placed into a slit made in a rubber stopper, and inserted into a glass cell (a modified version of a T-cell) [37,38,39]. 3 µL of RTIL was drop-cast on the unmodified and Pt-deposited MATFEs. Prior to the introduction of oxygen, the cell was purged with nitrogen gas to remove dissolved gases and atmospheric impurities. When the baseline was stable (after ca. 20 min), oxygen gas was introduced into the cell and continuously flowed over the electrode. To obtain different concentrations of oxygen, 100% oxygen gas was diluted with nitrogen through a gas mixing system, as reported by Lee et al. [39], which consisted of two digital flow meters (0–1.0 L/min, John Morris Scientific, NSW, Australia), one connected to an oxygen gas cylinder (O_2_) and the other to a nitrogen cylinder, using PTFE tubing via a Swagelok T-joint (Swagelok, Kardinya, WA, Australia). The O_2_/N_2_ mixture was then passed through an additional gas-mixing segment [39] to increase turbulence, and to ensure adequate mixing of both gases. The relative flow rates were used to calculate the different concentrations of oxygen gas introduced into the T-cell, with the sum of the flow rates kept constant at 800 cm^3^/min.

### 2.4. Electrode Imaging

MATFE surfaces were imaged via scanning electron microscopy (SEM), taken on a Zeiss Evo 40XVP microscope, at an accelerating voltage of 5.0 kV. The images were obtained at a working distance of 7 mm, with an aperture size of 30 µm, by combining two signals SE1 and SE2, which were detected by InLens and SE detectors, respectively. A thin coating of platinum was deposited over the electrodes due to the charging from the SU-8 polymer layer by the electron beam.

## 3. Results

### 3.1. Deposition and Characterization of 3D Nanostructured Microarrays

To increase the surface area of the commercially-available microelectrode arrays, the recessed microholes were over-filled with platinum to create high surface area three-dimensional structures with regular spacing. Eight depositions were performed in 0.5 M H_2_SO_4(aq)_, varying the following parameters: total deposition time (300 s or 420 s), number of steps (1 or 2), and composition of the platinum plating solution (with and without lead acetate). The platinum salt concentration (20 mM), lead acetate concentration (2 mM), and deposition potential (overpotential region, −0.2 V vs. Ag/AgCl) were fixed to observe the effect of the presence of lead acetate on the structure and shapes of the electrodeposits obtained. Cyclic voltammograms for some of the deposition processes in H_2_PtCl_6_/H_2_SO_4_ were included in our previous work [18]. The different parameters employed are given in detail in the experimental section, and their abbreviations are given in the first column in Table 2.

**Figure 1:**
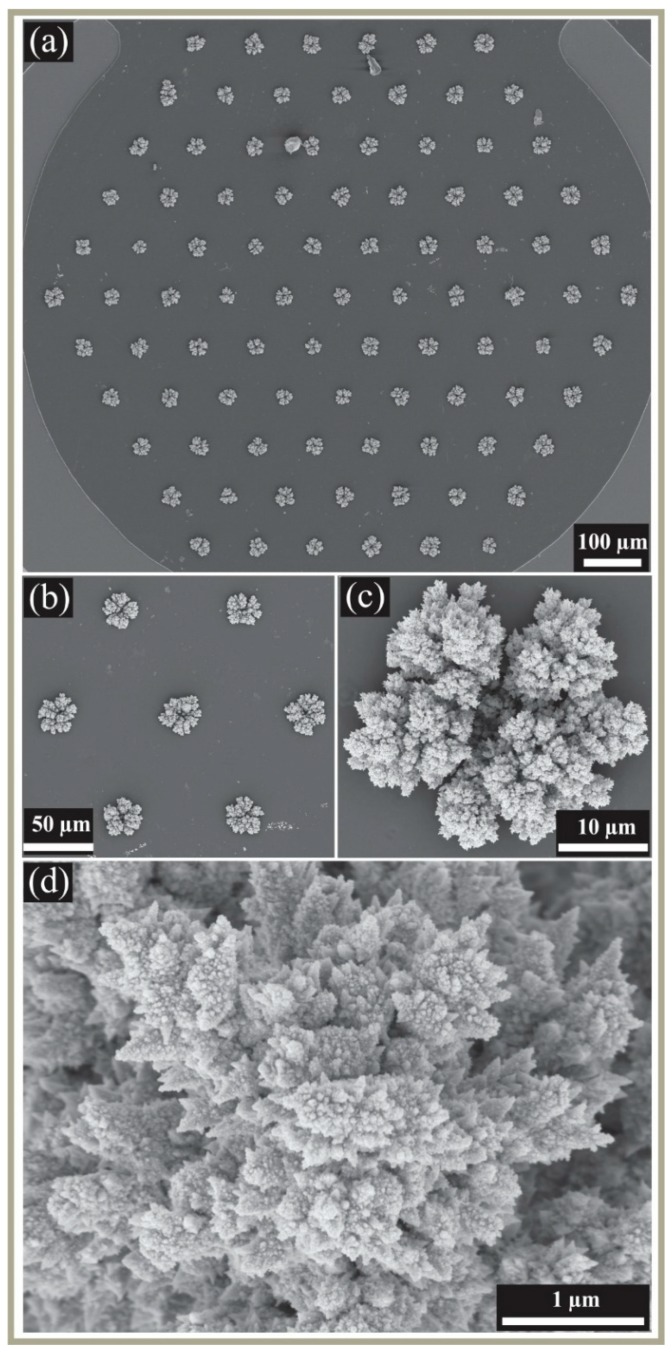
Scanning electron microscopy (SEM) images for Pt 3D nanostructure decorated Microarray thin-film electrodes (MATFEs) using 300 s deposition at −0.2 V from 20 mM H_2_PtCl_6_ in the presence of 2 mM Pb(OAc)_2_, showing (**a**) the whole electrode (90 electrodes, with deposits of diameter 27 ± 0.6 µm), (**b**) zoomed-in image of seven deposits, (**c**) single dendritic shaped 3D nanostructure, and (**d**) zoomed-in image showing the fine structural detail.

All depositions resulted in the formation of 3D structured deposits of platinum into and over the top of the microholes. All structures appeared to be strongly bound to the substrate and did not detach from the surface after vigorous rinsing with water or under a strong flow of air, which was performed to remove excess electrolyte before imaging, and to dry the electrode, respectively. Figure 1 shows scanning electron microscopy (SEM) images of the electrodeposits obtained from a single-step 300 s deposition in 20 mM H_2_PtCl_6_, in the presence of 2 mM Pb(OAc)_2_: (a) The whole electrode with 90 filled microholes, (b) a zoomed-in image showing seven adjacent deposits, (c) a single deposit, and (d) a close-up image of the fine structure of one electrodeposit. This particular deposition results in discrete, dendritic shaped nanostructures with star like spikes at the top, decorated with small nanoparticles. This sharp and spiky structure is likely to have a significantly higher electroactive surface area, compared to the recessed electrode, and also a larger surface area, compared to the deposits obtained in the absence of Pb(OAc)_2_, as discussed later.

**Figure 2:**
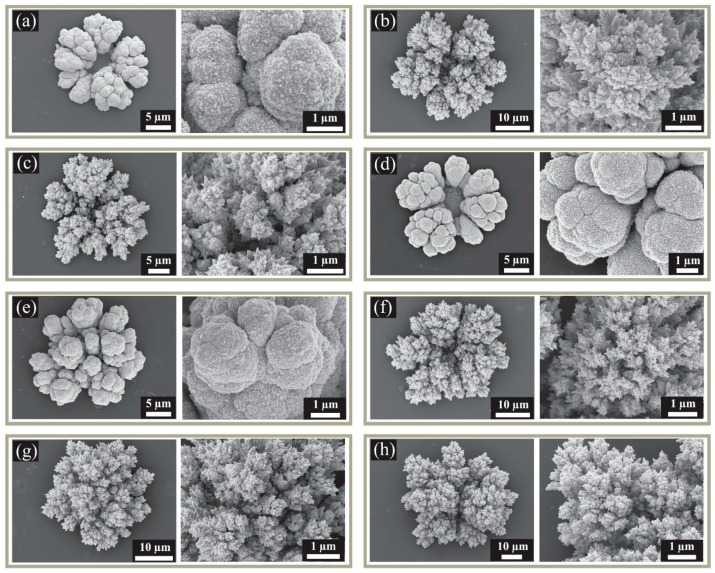
Scanning electron microscopy (SEM) images for the different nanostructured MATFEs with corresponding zoomed-in images showing the fine detail. (**a**) 300 s Pt, (**b**) 300 s Pt/Pb, (**c**) 150 s Pt/Pb + 150 s Pt/Pb, (**d**) 420 s Pt, (**e**) 300 s Pt + 120 s Pt, (**f**) 420 s Pt/Pb, (**g**) 300 s Pt + 120 s Pt/Pb, (**h**) 300 s Pt/Pb + 120 s Pt/Pb.

Images were also taken of the deposits obtained using the different deposition parameters. Figure 2 shows an image of the 3-D deposit observed at a single microhole (left), and a close-up view of that structure (right), for all eight deposition parameters employed in this work. For the single-step deposits (Figure 2a,b,d,f), the deposits appear to have less material in the center of the structure. This is due to the greater contribution of (slower) linear diffusion within the pores, compared to the (faster) radial diffusion at the edges, resulting in more deposits growing over the edges [18]. In a two-step deposition, this hole becomes filled probably as a result of many new nucleation sites occurring in this region; this is demonstrated in Figure 2d (one-step process) and 2e (two-step process), which both use a 420 s total deposition time. The extra material filling the center of the structure in Figure 2e is likely to lead to increased flux of analyte species, as a result of more multi-dimensional diffusion and accessible platinum sites. All of the deposits obtained from the pure platinum plating solution (H_2_PtCl_6_ in H_2_SO_4(aq)_) are relatively smooth in the close-up images (Figure 2a,d,e).

When lead acetate was added to the plating bath, dendritic structures were formed. Dendritic growth can be regarded as a competition between the order associated with crystal symmetry and the instabilities that are induced in the system during the growth process. For the case of Pt deposition in the presence of lead acetate, the latter is a foreign species which contributes to such instabilities when the Pt is being electrodeposited. Previous work [26] using a similar plating bath composition, indicated that Pb underpotential deposition (UPD) occurs during the growth process. The presence of Pb as the foreign species competes with Pt deposition, and therefore perturbs its growth. The Pb creates pinning centers, as it is the metal at the lower concentration, thereby inducing disorder and dendritic growth.

Using the same deposition time as Figure 2a, structures with much rougher and spiky surfaces are present in Figure 2b. There is also evidence of the “hole” in the center of the single step processes (Figure 2b,f), although it is not as obvious as the equivalent non-dendritic structures (Figure 2a,d), probably due to the formation of dendritic sites in all directions that partially fill the hole. In the two-step depositions at longer times (Figure 2g,h), this hole becomes filled, leaving a spiky, high surface area 3D-shaped deposit protruding over the microhole. The shape of the structure is quite similar regardless of whether the first step was dendritic or non-dendritic in nature, since the second step produced additional dendritic structures on the top of the initial electrodeposited structure. However, the size (diameter) of the deposit appeared to be larger when both steps were performed in the presence of lead acetate.

The diameters of the structures were measured at their widest point and are included in Table 2. Some structures (e.g., Figure 2d,e,f) appear slightly more elongated than others, so these values are merely a guide to the approximate relative dimensions of the deposits. Overall, the diameters of the deposits were wider for the dendritic structures, likely leading to a higher surface area of available Pt sites. It is difficult to judge the improvements simply by measurement of the diameter, so the increased electroanalytical abilities of these surfaces can be accurately determined by the calculation of the electroactive surface area, as described in the next section.

### 3.2. Electroactive Surface Area Calculation

**Figure 3:**
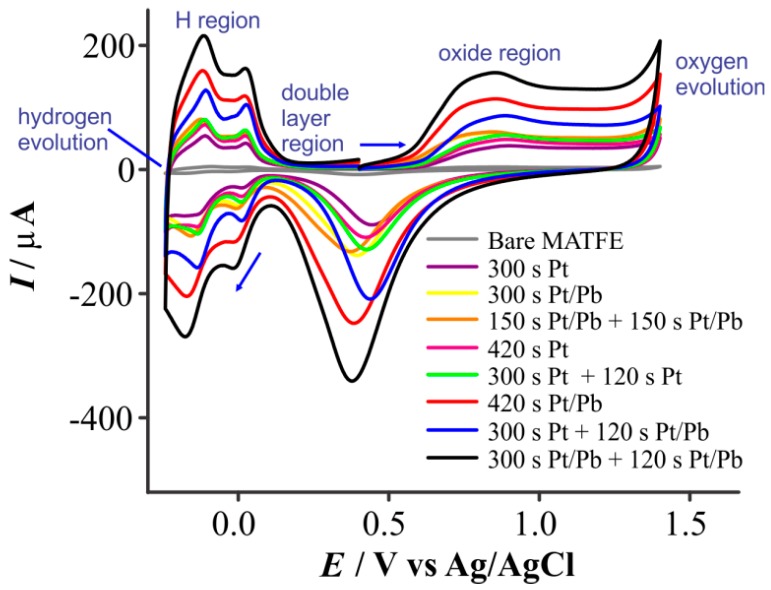
Cyclic voltammograms (10th cycle, 500 mVs^−1^) recorded on the different MATFEs between +1.4 and −0.24 V in a N_2_-saturated solution of 0.5 M H_2_SO_4_. The integrated area (*Q* = *I* × *t*) of the H_2_ desorption peaks (background subtracted) was used to calculate the electroactive surface area (ESA).

Given the rough nature of the deposits obtained from electrodeposition, the electroactive surface area (ESA) of each electrode was calculated using cyclic voltammograms (CVs) recorded in 0.5 M H_2_SO_4(aq)_. Figure 3 shows CVs in 0.5 M H_2_SO_4_ for all nine surfaces used in this study, scanned between the oxygen evolution and hydrogen evolution potentials, showing the characteristic behavior observed on platinum electrodes in H_2_SO_4_ [40,41]. There are three main potential regions: (1) Above 0.5 V, oxidation processes corresponding to the formation of adsorbed oxygen, ‘oxide region’, (2) the small region where little current is observed, the ‘double layer region’, and (3) the region below 0.2 V, where two couples of reversible hydrogen adsorption/desorption processes are observed, ‘H region’ [40,41]. The two processes in the H region are from weakly and strongly adsorbed hydrogen on the different crystal facets of Pt [40].

The charge under the hydrogen desorption peaks in Figure 3 was measured, and the ESA was calculated using standard methods and assuming a monolayer coverage of hydrogen of 210 µC/cm^2^ [35,36]. The ESA values have been included in Table 2 and show a huge increase from 1.01 mm^2^ for the recessed MATFE up to 44.1 mm^2^, for the largest structure where a two-step deposition in the presence of lead acetate (300 s + 120 s) was used to form the 3D structures. The other deposition parameters resulted in ESAs in-between these two values, consistent with the order of the approximate diameters of the structures. The shape of the hydrogen desorption region from −0.24 to 0.25 V was consistent amongst all samples, where the peaks at ca. −0.15 and 0.05 V were for Pt(110) and Pt(100) crystal facets, respectively [42]. Therefore, any differences in sensing performance at these electrodes could not be attributed to differences in crystallographic orientation of the surface.

### 3.3. Electrochemical Reduction of Oxygen on the Prepared Electrodes

The prepared electrode arrays were employed as working electrodes for oxygen reduction in a room temperature ionic liquid (RTIL). Oxygen reduction typically proceeds via a chemically reversible one-electron process in aprotic RTILs, as shown below [43,44]:

O_2_ + *e*^−^ ⇌ O_2_^∙−^, (1)

The RTIL chosen for this study was octyltriethylammonium bis(trifluoromethylsulfonyl)imide ([N_8,2,2,2_][NTf_2_]), since oxygen reduction voltammetry in ammonium RTILs gives a clear one-electron steady-state reduction wave well separated from the second reduction wave (reduction of superoxide to peroxide) [43,45]. It also gives a transient (peak-shaped) oxidation peak on a microdisk electrode, due to the large disparity in the diffusion coefficients of oxygen and superoxide [43,45].

**Figure 4:**
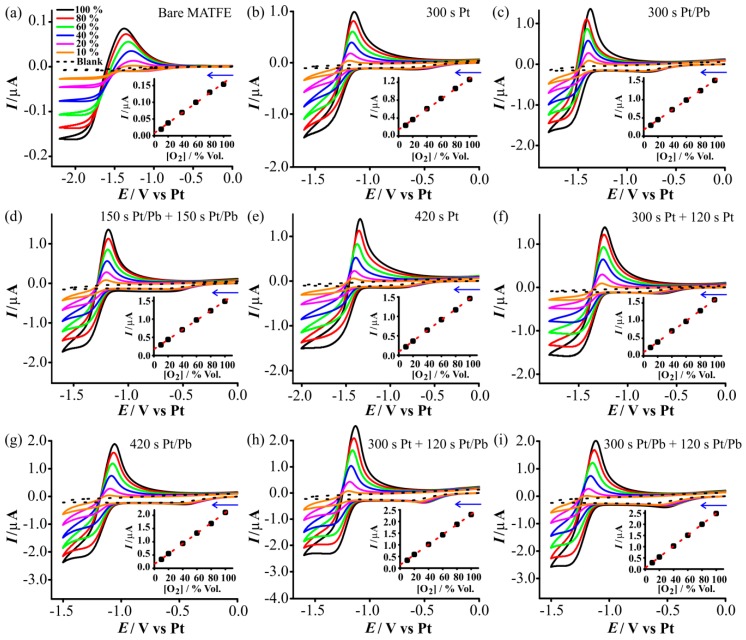
Cyclic voltammetry (CV) at 100 mVs^−1^ for the reduction of oxygen (10–100% vol.) in [N_8,2,2,2_][NTf_2_] on the different prepared MATFEs: (**a**) recessed, (**b**) 300 s Pt, (**c**) 300 s Pt/Pb, (**d**) 150 s Pt/Pb + 150 s Pt/Pb, (**e**) 420 s Pt, (**f**) 300 s Pt + 120 s Pt, (**g**) 420 s Pt/Pb, (**h**) 300 s Pt + 120 s Pt/Pb, (**i**) 300 s Pt/Pb + 120 s Pt/Pb. The dashed line is the response in the absence of oxygen. Currents on all surfaces were measured from a suitable potential, where the voltammetry showed steady state currents. The insets show the calibration plots of peak current (baseline corrected) vs. concentration, along with the line of best fit.

Figure 4 shows CVs for the reduction of oxygen at concentrations between 10 and 100% vol. O_2_ (gas phase) in the RTIL [N_8,2,2,2_][NTf_2_], at a scan rate of 100 mVs^−1^. The blank scan in the absence of oxygen is shown as the dotted line. On the bare MATFE (Figure 4a), the CV shape is characteristic of oxygen reduction on a microdisk electrode using a structurally similar ammonium RTIL, [N_6,2,2,2_][NTf_2_] [43,45]. When the electrode surface was modified with 3D structures, the separation between the forward and reverse scans became slightly wider and the currents become larger, but the overall shape of steady-state reduction and peak-shaped oxidation remained the same. The small reductive feature prior to the main oxygen reduction process is likely to be an impurity in the ammonium RTIL, since this feature was also present in the blank scans (dotted lines in Figure 4). This gets slightly larger as the ESA of the electrode gets larger, therefore the background current was subtracted from all analyte currents to exclude this feature. The calibration graphs (maximum current vs. concentration) are shown in the insets to Figure 4 and are all highly linear (R^2^ > 0.999), showing excellent analytical performance on all electrodes. The sensitivities (gradients) and LODs obtained from analyzing the lines of best fit are given in Table 2 and will be discussed later.

The reference electrode built-in to the MATFE was made from platinum, which is prone to potential shifting depending on the nature (cleanliness) of the surface, with potential shifts of up to 200 mV observed in the presence of 20% O_2_ [46]. Therefore, to properly calibrate the potential for oxygen reduction, ferrocene was added at the end of the calibration experiment, and CVs were recorded for the ferrocene/ferrocenium (Fc/Fc^+^) redox couple (10 mM Fc), together with the oxygen/superoxide redox couple (100% O_2_). The half-wave potential (*E*_1/2_) for oxygen reduction was measured relative to the mid-point of Fc/Fc^+^, and calculated so that Fc/Fc^+^ was at 0 V. The last column of Table 2 shows that *E*_1/2_ for oxygen reduction did not change (within experimental variation) for the modified electrodes but is about 100 mV shifted from the unmodified MATFE. It is possible that the fresher Pt deposits may be more catalytic in nature, which could account for this shift.

**Figure 5:**
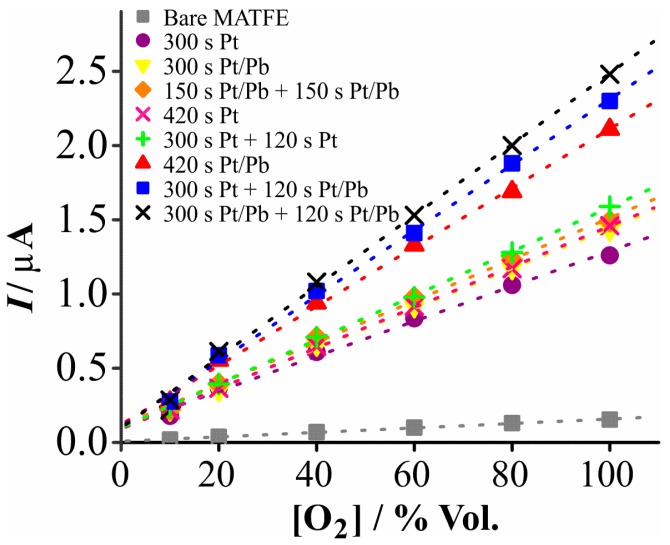
Plots of absolute current vs. oxygen concentration for the reduction of 10–100% vol. oxygen in [N_8,2,2,2_][NTf_2_] on: a recessed MATFE (■), 300 s Pt (●), 300 s Pt/Pb (▼) 150 s Pt/Pb + 150 s Pt/Pb (♦), 420 s Pt (×), 300 s Pt + 120 s Pt (+), 420 s Pt/Pb (▲), 300 s Pt + 120 s Pt/Pb (■), 300 s Pt/Pb + 120 s Pt/Pb (×) deposited structures.

Figure 5 shows a plot of the calibration data for all eight modified surfaces, compared to the unmodified MATFE (grey squares). Clearly, a significant sensitivity enhancement for oxygen reduction was achieved by modification of the MATFEs. It is noted that we previously used the 300 s Pt deposition ‘cauliflower’, for improved detection of ammonia in RTILs [18]. This particular deposit is shown for oxygen reduction as the purple circles in Figure 5. Although this already gives a significant sensitivity enhancement compared to the unmodified MATFE, it has the lowest sensitivity compared to the other modified structures, clearly showing the significant benefits associated with the formation of dendritic structures, extended times, or two-step depositions.

Focusing on the five MATFEs with a total deposition time of 420 s, the greatest sensitivity improvements were observed when Pb(OAc)_2_ was present in at least one of the plating bath solutions. The two deposits obtained in the absence of Pb(OAc)_2_ (shown as green plus signs and pink crosses in Figure 5, with total time = 420 s), had sensitivity values closer to those of the 300 s depositions in Pt/Pb, clearly showing the benefit of adding lead acetate to the plating bath solution. Overall, the two-step depositions showed higher sensitivities compared to one-step depositions with the same total deposition time, likely due to the filling of the holes and the creation of additional 3D structures above the microhole, as observed in the SEM images in Figure 2.

Table 2 shows the sensitivities obtained from the gradients of the best fit lines, along with the other analytical parameters (highest peak current, limit of detection). The highest sensitivity was ca. 16 times larger, compared to the unmodified MATFE. This is a clear and significant improvement but it does not reflect the ca. 44 times increase in the ESA. This could be due to partial overlap of diffusion zones at the modified structures; the commercial devices are designed to be separated by 10 times the diameter (*d*) to their neighboring electrodes. The deposited structures were approximately three times larger than the original electrode diameter, suggesting that there is only ~3.3 times *d* separation from the adjacent electrodes. This is likely to result in overlapping of the diffusion zones on the timescale of the CV experiment [12]. Another reason for the lower than expected sensitivity increase could be due to some loss of ESA upon exposure to the RTIL, which will be discussed later in Section 3.4. The higher sensitivity leads to lower LODs, showing an improvement from 5.8% O_2_ (unmodified electrode) to 1.5% O_2_ (300 s Pt/Pb + 120 s Pt/Pb). We note that much lower LODs are possible by studying a smaller concentration range, but that was not required in this work.

The reproducibility of all modified and unmodified MATFEs was excellent for different additions of the same concentration of O_2_ gas (~2–3% current variation), such that the error bars were very small and hard to see on the figure. However, a ca. 10–15% variation in current was observed when several electrodes (*n* = 5) modified using the same deposition parameters were employed, representing the slightly different nucleation sites possible with each new deposition step. Therefore, it is recommended that each electrode is calibrated before use with gas standards for accurate quantification of O_2_ gas in an unknown environment. Overall, this is a relatively small variation given the significant current enhancement made possible by the electrode modification.

### 3.4. Loss of Electroactive Surface Area after Experiments in Ionic Liquids

**Figure 6:**
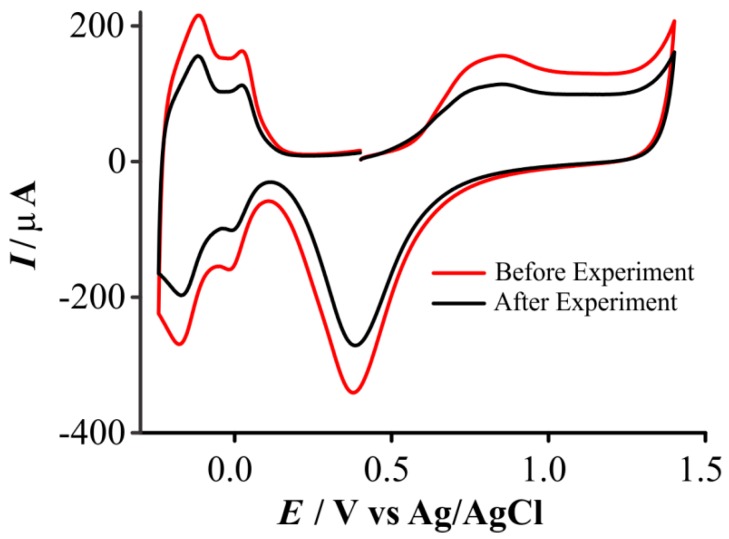
Comparison of cyclic voltammograms (10^th^ cycle, 500 mVs^−1^) recorded before (red) and after (black) a gas sensing experiment in the room temperature ionic liquid (RTIL) on a 3D nanostructure decorated MATFE (300 s Pt/Pb + 120 s Pt/Pb) in N_2_-saturated 0.5 M H_2_SO_4(aq)_.

During the calculation of electroactive surface area (ESA), it was observed that the charge under the hydrogen adsorption/desorption peaks was smaller after experiments in ionic liquids had been performed. This is demonstrated in Figure 6, which shows CVs in sulfuric acid for a freshly prepared deposit (red line, in this case, 300 s deposition in Pt/Pb followed by 120 s in Pt/Pb), compared to the same electrode after oxygen reduction experiments (black line). There was an ~40% loss in electroactivity after oxygen reduction experiments in RTILs. Upon further investigation, we believe that this was not simply caused by scanning of potential in the oxygen reduction region, since a loss of ~25% activity occurred upon exposure to the ionic liquid with no potential scans performed. This is an interesting observation and warrants further studies to fully understand this phenomenon. Since we also observed this in other experiments (e.g., for ammonia oxidation in RTILs, experiments not shown), it is important that this effect be considered when assuming electroactive surface areas for electroanalytical studies in RTIL solvents. The accepted behavior of Pt in acidic aqueous solutions may not necessarily translate to RTIL solvents, since the charge distribution at the electrode surface was likely to be different a conventional solvent/electrolyte system. It is known that Pt is prone to dissolution in aqueous and organic solutions during the oxygen reduction reaction [47], however there is clearly a different phenomenon when ionic liquids are employed, given the decrease in the ESA measured after simple immersion of the electrode in the RTIL without any applied potential. This suggests that there may be residual RTIL present on the surface of the Pt electrode which is difficult to remove by a simple washing procedure, which blocks some of the electrochemically active sites.

## 4. Conclusions

Platinum 3D structures were obtained via electrodeposition into the holes of recessed microelectrode arrays. The number of steps involved in the deposition process was varied (one or two), along with the total time for deposition (300 s or 420 s). A single step deposition produced structures with a hole (i.e., less deposit) in the middle, but the hole was mostly filled during the second deposition process. With only the platinum complex present in the plating bath, the platinum structures were relatively smooth and resembled the buds of a cauliflower. When Pb(OAc)_2_, was added to the plating bath, dendritic structures were formed. The highest surface area was observed for two-step deposition with a total time of 420 s, with Pb(OAc)_2_ present in both steps. This surface showed sensitivity enhancements of 16 times that of the recessed electrode towards oxygen reduction. These enhancements were lower than expected given the increased ESA (44 times), and this is attributed to (i) overlapping diffusion profiles because of the larger structures, and/or (ii) a loss in some electroactivity upon exposure to the ionic liquid. Overall, we have demonstrated significantly enhanced sensitivity towards oxygen reduction by the formation of dendritic 3-D nanostructured materials, providing an avenue to modify microarray (and other) electrodes for enhanced electroanalytical responses, for the improved detection of analyte species.

## Figures and Tables

**Table 1:** Description of the different deposition conditions, plating bath compositions, and deposition times used in this work. All solutions were in a background of 0.5 M H_2_SO_4(aq)_.

Abbreviation	Deposition Conditions and Plating Bath Composition	Deposition Time
300 s Pt	Single step, 20 mM H_2_PtCl_6_	300 s
300 s Pt/Pb	Single step, 20 mM H_2_PtCl_6_ + 2mM Pb(OAc)_2_	300 s
150 s Pt/Pb + 150 s Pt/Pb	Two steps, both in 20 mM H_2_PtCl_6_ + 2mM Pb(OAc)_2_	150 s + 150 s = 300 s
420 s Pt	Single step, 20 mM H_2_PtCl_6_	420 s
300 s Pt + 120 s Pt	Two steps, both in 20 mM H_2_PtCl_6_	300 s + 120 s = 420 s
420 s Pt/Pb	Single step, 20 mM H_2_PtCl_6_ + 2mM Pb(OAc)_2_	420 s
300 s Pt + 120 s Pt/Pb	Two steps. First step: 20 mM H_2_PtCl_6_, second step: 20 mM H_2_PtCl_6_ + 2mM Pb(OAc)_2_	300 s + 120 s = 420 s
300 s Pt/Pb + 120 s Pt/Pb	Two steps, both in 20 mM H_2_PtCl_6_ + 2mM Pb(OAc)_2_	300 s + 120 s = 420 s

**Table 2:** Analytical parameters obtained: diameter of deposit, charge (*Q*_H_), electroactive surface area (ESA, calculated from the integration of H_2_ desorption peak obtained in N_2_-saturated 0.5 M H_2_SO_4_ at 500 mVs^−1^ vs. Ag/AgCl RE and Pt CE before gas experiments), reduction peak current (*I*_p_) for 100% vol. oxygen, sensitivity, and limit of detection (LOD) calculated for oxygen reduction (10–100% vol.), on all electrodes using cyclic voltammetry in the RTIL [N_8,2,2,2_][NTf_2_]. Half-wave potential (*E*_1/2_) for oxygen reduction is reported vs. the ferrocene/ferrocenium (Fc/Fc^+^) redox couple.

Deposition Method	Diameter of Deposit/µm	*Q*_H_ = *I* × *t*/(µC)	ESA/(mm^2^)	*I*_p_ (100% O_2_)/(µA)	Sensitivity/(nA/%vol.)	LOD^2^/(% vol.)	*E*_1/2_ vs. Fc/Fc^+^/V
Bare MATFE	n/a	2.13	1.01	0.15	1.5	5.8	−1.45
300 s Pt	22 ± 0.8 ^1^	21.7	10.3	1.26	14.1	4.5	−1.35
300 s Pt/Pb	27 ± 0.6	31.6	15.0	1.43	13.7	2.4	−1.37
150 s Pt/Pb + 150 s Pb/Pb	25 ± 0.7	32.8	15.6	1.49	13.7	2.1	−1.35
420 s Pt	28 ± 0.8	28.9	13.8	1.46	13.9	2.3	−1.35
300 s Pt + 120 s Pt	30 ± 0.8	32.3	15.4	1.59	15.1	2.4	−1.34
420 s Pt/Pb	36 ± 0.9	68.1	32.4	2.11	19.8	2.4	−1.34
300 s Pt + 120 s Pt/Pb	37 ± 2.1	53.7	25.6	2.30	22.1	2.1	−1.31
300 s Pt/Pb + 120 s Pt/Pb	52 ± 4.2	93.3	44.4	2.48	24.3	1.5	−1.34

^1^ Error bars represent one standard deviation of seven 3D nanostructure deposits on the same microarray. ^2^ The LODs are calculated from 3 standard deviations of the line of best fit, in the range 10–100% O_2_, and serve merely as a comparison of the different surfaces. Lower LODs are possible if a different concentration range is studied but it is not the focus of this work.
